# The Effect of Oncology Nurse Navigation on Mental Health in Patients with Cancer in Taiwan: A Randomized Controlled Clinical Trial

**DOI:** 10.3390/curroncol31070306

**Published:** 2024-07-20

**Authors:** Wei-Zhen Yu, Hsin-Fang Wang, Yen-Kuang Lin, Yen-Lin Liu, Yun Yen, Jacqueline Whang-Peng, Tsai-Wei Huang, Hsiu-Ju Chang

**Affiliations:** 1 School of Nursing, College of Nursing, Taipei Medical University, Taipei 110301, Taiwan; d432104005@tmu.edu.tw; 2 Taipei Cancer Center, Taipei Medical University, Taipei 110301, Taiwan; 116096wang@gmail.com (H.-F.W.); ylliu@tmu.edu.tw (Y.-L.L.); jqwpeng@nhri.org.tw (J.W.-P.); 3 Graduate Institute of Athletics and Coaching Science, National Taiwan Sport University, Taoyuan 333325, Taiwan; robbinlin@ntsu.edu.tw; 4 Pediatric Oncology, Department of Pediatrics, Taipei Medical University Hospital, Taipei 110301, Taiwan; 5 Department of Pediatrics, School of Medicine, College of Medicine, Taipei Medical University, Taipei 110301, Taiwan; 6 Program for Cancer Biology and Drug Discovery, College of Medical Science and Technology, Taipei Medical University, Taipei 110301, Taiwan; yyen@tmu.edu.tw; 7 Research Center in Nursing Clinical Practice, Department of Nursing, Wan Fang Hospital, Taipei Medical University, Taipei, 116079, Taiwan; 8 Department of Nursing, College of Nursing, National Yang Ming Chiao Tung University, Taipei 112304, Taiwan; 9 Efficient Smart Care Research Center, College of Nursing, National Yang Ming Chiao Tung University, Taipei 112304, Taiwan

**Keywords:** neoplasms, oncology nursing, randomized controlled trials, mental health, psychological distress, anxiety, depression, demoralization

## Abstract

In this study, we investigated the prevalence of mental health problems among patients with cancer and whether oncology nurse navigation improved their mental health outcomes and medical experience. In this randomized controlled clinical trial, we recruited 128 outpatients with cancer via purposive sampling from a teaching hospital in northern Taiwan. Participants were randomly assigned to the navigation group (*N* = 61) or the usual care group (*N* = 67). Data were collected from January 2019 to July 2020 using questionnaires, including the self-reported Distress Thermometer, Hospital Anxiety and Depression Scale, Demoralization Scale, and Patient Assessment of Chronic Illness Care. Data were collected at baseline and after three and six months of the intervention. Descriptive and analytical statistical analyses were performed. The prevalence rates of anxiety, depression, distress, and demoralization were 17.9%, 15.7%, 29.7%, and 29.7%, respectively. After three months, the participants in the navigation group exhibited significantly reduced levels of anxiety, demoralization, and emotional distress (reduced by 92%, 75%, and 58%, respectively) and reported a better medical experience (odds ratio = 1.40) than those in the usual care group.

## 1. Introduction

According to the GLOBOCAN 2020 global cancer update, Asia has higher rates of cancer-related mortality (58.3%) and incidence (49.3%) than other regions [1]. It is estimated that by 2040, the cancer burden in low- and middle-income countries will increase from 12 million to 20 million cases per year [2]. While advances in diagnostic methods and medical treatment have extended the survival time of many patients with cancer to >5 years, it is common in Taiwan for patients with cancer to die from noncancer causes [3]. Therefore, the management of cancer should focus on both prolonging life and closely monitoring the mental health and quality of life of patients [4]. The psychological impacts of the initial diagnosis of cancer, uncertain course of treatment, adjustment to treatment side effects, financial burden, and possibility of disease recurrence lead to the development of mental health problems in many patients. Research has found that the incidence of mental problems is markedly increased in patients suffering from cancer [4,5,6]. The management of cancer must involve treatment of the physical disease, as well as assessment and care of the psychosocial impact on patients and their families [7].

An oncology nurse navigator (ONN) assists with the implementation of policies for the management of cancer, including prevention, early detection, diagnosis, treatment, quality of life, and survival of cancer care. This role is important for improving the mental health of patients [8,9,10,11]. An ONN is defined by the Oncology Nursing Society (ONS) as “a professional RN [registered nurse] with oncology-specific clinical knowledge who offers individualized assistance to patients, families, and caregivers to help overcome healthcare system barriers. Using the nursing process, an ONN provides education and resources to facilitate informed decision-making and timely access to high-quality health and psychosocial care throughout all phases of the cancer continuum” (p. 4) [12,13]. Duties include building empathic and trusting relationships with cancer patients through initial phone calls prior to their clinic visits, providing them with immediately accessible resources, and focusing on overcoming communication barriers between them and their healthcare providers regarding mental health [10,14].

The benefits provided by an ONN during the entire disease journey of the patient have been extensively documented in the literature [9]. The effectiveness of ONN interventions in terms of the timeliness of screening, treatment completion rate, quality of life, and satisfaction is excellent [15,16,17]. Other studies have also revealed high levels of satisfaction with ONN intervention [15,18]. However, only a few studies thus far have found that navigation care can improve aspects of mental health such as emotional distress, depression, anxiety, self-efficacy, quality of life, and emotional support [18,19,20,21]. Although demoralization and depression are completely different mental health problems, they are strongly correlated with each other in patients with cancer. Demoralization is also strongly correlated with suicidal ideation and can be used as a predictor of depression among such patients [22]. The currently available literature on the psychosocial effects of ONNs on the cancer continuum is insufficient. Thus, further in-depth investigation is required in this field.

Despite the recognized benefits of ONNs, the currently available literature on the psychosocial effects of ONNs on the cancer continuum is insufficient. There is a notable knowledge gap regarding the impact of ONNs on specific mental health aspects, such as medical experience, demoralization, and distress. Furthermore, nurse navigation is rarely implemented in Taiwanese medical institutions [23].

Therefore, the purpose of this study was to address this gap by evaluating the prevalence of depression, anxiety, discouragement, and distress among patients with cancer. Our research question was as follows: Does the implementation of an oncology nurse navigator program improve the mental health outcomes and medical experience of patients with cancer? We hypothesized that patients receiving ONN support would exhibit significantly lower levels of anxiety, depression, demoralization, and distress and report a better medical experience compared with those receiving usual care.

## 2. Materials and Methods

### 2.1. Research Design

The study consisted of a randomized controlled clinical trial to evaluate the impact of nurse navigators on the mental health of cancer patients. The primary outcomes were mental health indicators (depression, anxiety, demoralization, and distress) at a posttest (three months after the baseline) and follow-up (six months after baseline), and the secondary outcome was the patient’s experience of cancer care at a posttest and follow-up.

### 2.2. Participants and Setting

The participants were outpatients recruited from the cancer center of a regional teaching hospital in northern Taiwan from January 2019 to July 2020. Patients who met the following criteria were included: age ≥ 20 years, diagnosed with cancer regardless of location, receiving active cancer treatment (such as chemotherapy, radiotherapy, hormone therapy, immunotherapy, or targeted therapy), ability to speak Chinese, and willingness to participate and provide written informed consent. Exclusion criteria included preexisting psychological problems and patients receiving palliative or hospice care. These criteria were verified through medical record reviews and assessments by ONNs. The purpose of this study was to evaluate the effectiveness of nurse navigation intervention, specifically in terms of its psychosocial impact. Patients with mental illness before their cancer diagnoses could not be distinguished from patients who developed mental problems after their cancer diagnoses, which would affect the analysis of the effects of navigation. Furthermore, patients receiving palliative medical care were excluded as this was not covered in the navigation service of the study site.

### 2.3. Randomization and Blinding

We used the RAND function in Excel (Microsoft Office 2016) to generate a random number table for the random allocation of participants to groups. We divided the participants equally (allocation ratio of 1:1) into two groups; the navigation group received the nurse navigator care and case management (experimental, *N* = 61), and the usual care group received only case management (control, *N* = 67). This study was a single-blinded trial; while it was not possible to blind the researchers, the participants were blinded. The staff who provided the usual care in the hospital were also blinded to the group allocation. Administrative assistants were responsible for collecting patient questionnaires. After the study was completed, the ONNs also provided the navigation care intervention to the usual care group.

### 2.4. Intervention

**Figure 1:**
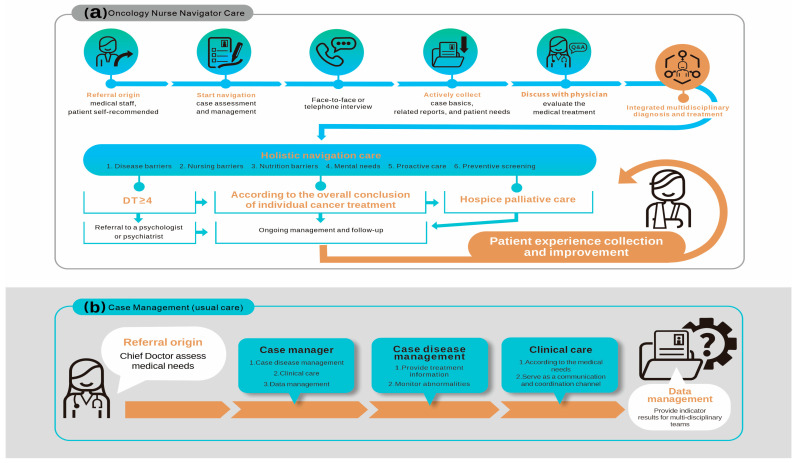
Intervention flow-chart. (**a**) Oncology nurse navigator care. (**b**) Case management (usual care).

In the navigation group, the “patient-centered” approach was adopted. Patients were referred by medical staff or self-recommended. Once referred, oncology nurse navigators (ONNs) initiated the navigation process with thorough case assessment and management, including face-to-face or telephone interviews to gather detailed patient information and actively collect case basics, related reports, and patient needs. This phase corresponds to the baseline measurement of primary and secondary outcomes. Following information collection, the ONNs discussed the medical treatment plan with physicians to evaluate and refine the approach. This collaborative effort led to integrated multidisciplinary diagnosis and treatment, ensuring a comprehensive and tailored care plan for each patient. Holistic navigation care was then provided, addressing various barriers including disease management, nursing, nutrition, mental needs, proactive care, and preventive screening. Patients received regular assessments and ongoing management, allowing the care plan to be adapted as needed. Specialized interventions were also available for patients experiencing high levels of distress (DT ≥ 4), including referrals to psychologists or psychiatrists. Ongoing management and follow-up assessments of outcomes were conducted three months and six months after the start of the intervention based on the overall cancer treatment plan, which included a provision for hospice palliative care when necessary. This timeline aligns with standard clinical practice for assessing intervention efficacy over time. Continuous collection and analysis of patient feedback were undertaken to enhance the quality of care. Additionally, the navigation group received nurse navigator care and case management, ensuring a structured and consistent approach throughout the treatment phases. The alignment of nurse navigator care and case management guaranteed that the collection of patient data remained consistent over time, regardless of the phase of treatment. This structured approach facilitated precise measurement of the outcomes at predefined intervals, specifically three and six months post-intervention, providing reliable data for assessing the effectiveness of the interventions (Figure 1).

The intervention service included at least two assessments per month for outpatient participants (for example, telephone follow-ups and outpatient interviews) to detect improvements in treatment response. Further, preclinical face-to-face interviews were arranged to evaluate the effectiveness of the patients’ self-management and ensure that appropriate assistance was provided throughout their upcoming hospital visits. Through this process, the patient was informed of the purpose of the outpatient visits and the medical plan to be followed thereafter. In addition, there was no limit to the number of times the patients could contact the nurse navigators using the electronic communication software. While the participants were in the hospital, their conditions were assessed twice weekly, and appropriate help was provided. The first visit for outpatient chemotherapy served to evaluate the appropriateness of treatment according to the patient’s needs, provide relevant health education before and after treatment (covering topics such as possible side effects and understanding the value of blood sampling), determine the patient’s specific needs, provide a 14 h (8:00 a.m.–10:00 p.m.) contact window, introduce the concept of second-opinion self-consultation, offer multidisciplinary medical care, and integrate domestic medical resources and the assistance and information provided by an international oncology expert, who was a provisional medical staff member from the USA collaborating with the research site in this study.

This intervention was provided by three clinical navigator nurses. The first was a senior nurse who had been engaged in cancer nursing for over 20 years. The second was a nurse who had worked in the cancer ward for over 5 years and was studying for a master’s degree in mental health at the time of the study. The third was a psychiatric mental health nurse with a master’s degree in nursing and more than 15 years of experience as a clinical nurse. In addition, the navigation team included three cancer specialist attending physicians and a nursing professor in the field of mental health, who was in charge of the nursing navigator care program.

Usual care with case management refers to the care service provided by the hospital under medical insurance benefits, and it adopts “tumor disease” as the main disease management model [24,25]. The services provided therefore mainly focused on treating patients according to cancer evaluation criteria, handling cancer clinical case management and follow-up-related tasks, providing emotional support and care for patients and their families following diagnosis and during treatment, developing and maintaining tumor databases and case management systems, assisting the patients with signing a do-not-resuscitate order, and integrating internal and external resources.

### 2.5. Instruments

#### 2.5.1. Sociodemographic and Disease-Related Characteristics

We obtained sociodemographic data (age, sex, marital status, occupation, education level, household monthly income, smoking status, drinking habits, use of areca (betel nut), life dependence, exercise habits, and religion) and disease-related data (time since diagnosis, cancer stage, cancer diagnosis, treatment regimen, hepatitis history, family history, comorbidities, and surgeries) from the patients’ records. Specifically, smoking status was defined as having smoked more than 100 cigarettes in one’s lifetime and having smoked in the last 30 days; drinking habits referred to almost daily consumption, with a pattern of drinking alone rather than socially; and the use of areca (betel nut) was characterized by daily use, with users experiencing discomfort if not consumed regularly. Exercise habits were defined as engaging in physical activity at least three times per week, with each session lasting at least 30 min, and reaching a heart rate of 130 beats per minute. Employment status indicated whether the participants were employed in a paid job; religion was recorded as having any current religious belief, irrespective of denomination; and comorbidities were noted as any chronic diseases present aside from cancer, such as hypertension, diabetes, or heart disease. The cancer stages in this study were categorized according to a number staging system into stages 0, I, II, III, and IV [26]. These stages were then grouped into three subsets: early, intermediate, and severe. The early group included stages 0 and I; the intermediate group included stages II and III; and the severe group included stage IV [27].

#### 2.5.2. Hospital Anxiety and Depression Scale (HADS)

The HADS contains two subscales: anxiety and depression (7 items per subscale for a total of 14 items). Each item is scored using a four-point Likert scale from 0 to 3, with higher scores indicating higher levels of anxiety or depression. Total scores < 7, from 8 to 10, and ≥11 indicate no anxiety or depression, suspected anxiety or depression, and the presence of anxiety or depression, respectively [28]. The Chinese version has also exhibited good validity, reliability, specificity, and sensitivity, and the Cronbach’s α values of the HADS and HADS-Depression were 0.88 and 0.75, respectively [29].

#### 2.5.3. Distress Thermometer (DT)

The DT was developed by Roth et al. [30] and introduced by the National Cancer Comprehensive Network as a simple and effective screening tool for distress symptoms [31]. Participants were instructed to circle their answers on a scale from 0 (no distress) to 10 (extremely distressed) to indicate their level of distress in the preceding week. A score ≥ 4 denoted high levels of distress [32]. The Chinese version of the DT has shown optimal sensitivity (0.78; 95% CI = 0.68~0.86), specificity (0.73; 95% CI = 0.65~0.80), test-retest reliability (r = 0.800, *p* < 0.001), and a Cronbach’s α value of 0.88 [33,34], and it is considered the sixth vital sign in oncology care [32,35].

#### 2.5.4. Demoralization Scale (Mandarin Version) (DS_MV)

The Demoralization Scale developed by Kissane et al. [36] comprises five subscales (loss of meaning, irritability, depression, helplessness, and sense of failure), with a total of 24 items. For the Mandarin version, the Cronbach’s α values of the total scale and the subscales were 0.853 and 0.720–0.894, respectively. The score of the DS_MV has good concurrent validity and divergent validity, and it is positively correlated with the Patient Health Questionnaire (PHQ) [37]. This scale uses a five-point Likert scale, with responses ranging from “strongly disagree” (0 points) to “strongly agree” (4 points). Here, a score ≥ 30 points indicates a high degree of demoralization [37].

#### 2.5.5. Patient Assessment of Chronic Illness Care (PACIC)

The PACIC scale was created to assess whether the care provided is consistent with the Chronic Care Model of the Center for Accelerating Care Transformation (ACT Center) in 2000, and it was designed to ensure that patients receive patient-centered care [38]. The scale comprises five subscales (patient activation, decision support, goal setting, problem-solving, and follow-up or coordination), with a total of 20 items. The Cronbach’s α values for the total scale and subscales were 0.93 and 0.77~0.90, respectively [39]. The scale has shown good construct and content validity [39].

### 2.6. Data Collection

**Figure 2:**
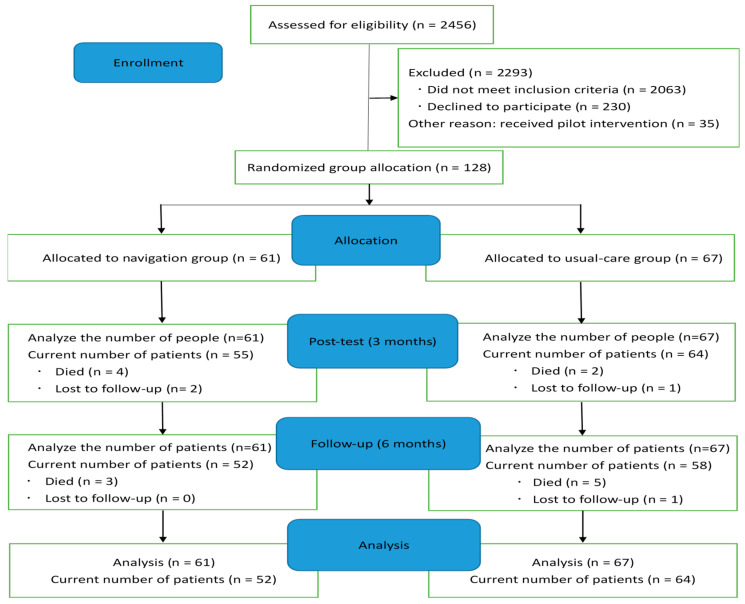
Study flow diagram.

We identified eligible patients by searching the hospital outpatient information system. The patients agreed to participate in the study during the initial telephone contact. On the day of their visit to the outpatient clinic, they provided written informed consent and completed the pre-test questionnaire. The collection of questionnaires was performed at three timepoints: baseline, three months later (posttest), and six months later (follow-up). Data collection was carried out and completed by two administrative assistants. Of the 2456 patients who were screened for eligibility, 393 met the inclusion criteria, and of those, 163 (41.5%) were enrolled in this study. Thirty-four patients received the pilot intervention, while the remaining 128 (61 in the navigation group and 67 in the usual care group) participated in this study (Figure 2). The completion rate was 85.9% ((128 − 14 − 3 − 1)/128). At the post-test timepoint, nine patients were lost (six in the navigation group and three in the usual care group). Another nine patients were lost at the follow-up timepoint (three in the navigation group and six in the usual care group). The reasons for this loss to follow-up are that 14 patients died, 3 patients withdrew from the study, and one was not physically able to fill out the questionnaires. Nonetheless, we used an intention-to-treat analysis, and these lost patients were thus included in the analysis.

### 2.7. Data Analysis

#### 2.7.1. Sample Size Estimation

G*Power version 3.1 and pilot study data were used to estimate the required sample size for our study [40]. For this purpose, we used the Distress Thermometer (DT) indicator (α = 0.05; standard deviation = 2.52; effect size = 0.21; navigation group: mean/*n* = 2.32/19; usual care group: mean/*n* = 3.4/15). The estimated minimum required sample size was 118 participants, and the power for post hoc testing was 0.83. Ultimately, 128 patients ensured the necessary power of 0.80.

#### 2.7.2. Statistical Methods

We analyzed the data using SPSS Statistics version 25 for Windows (IBM, Armonk, NY, USA). Missing values for 27 samples (mainly from the PACIC and DT questionnaires) were imputed using mean imputation. Analysis was conducted in two parts.

Firstly, we assessed the sociodemographic variables, disease-related variables, and primary outcome variables (demoralization, distress, anxiety, depression, and medical experience). The Kolmogorov–Smirnov test determined if the data followed a normal distribution. Normally distributed continuous variables were expressed as the mean ± standard deviation (SD), while non-normally distributed variables were expressed as the median with an interquartile range (IQR). Categorical variables were presented as counts and percentages. Independent *t*-tests were used to compare the normally distributed continuous variables between the two groups (navigation and usual care), while the Mann–Whitney U test was used for the non-normally distributed continuous variables. Fisher’s exact test was employed for categorical variables due to the relatively low number of observations.

The second phase focused on the effectiveness of the nurse navigation intervention. We used generalized estimating equations (GEEs) to examine the primary and secondary outcomes over three timepoints, analyzing time–group interactions. The GEEs included a first-order autoregressive correlation matrix (AR 1). Ordinal logistic regression with a cumulative logit link modeled the ordinal outcomes (anxiety and depression), logistic regression with a binomial family and logit link modeled the binary outcomes (demoralization and distress), and linear regression with a Gaussian family and identity link modeled the continuous variable (patient experience of cancer care).

Statistical significance was established at *p* values < 0.05 (two-sided), with 95% confidence intervals for all significant results. Feise [41] stated that multiple primary outcomes do not necessitate an additional type 1 error adjustment, given that type 1 and type 2 errors are mutually reinforcing.

### 2.8. Ethical Considerations

The study was approved by the Joint Institutional Review Board of a medical university in northern Taiwan (IRB number: N201809031) and registered at ClinicalTrials.gov (number: NCT05537870). All participants provided written informed consent after they received information regarding the benefits and risks of the study. Data were anonymized by assigning a number to each participant.

## 3. Results

### 3.1. Demographic and Instrument Characteristics

Of the 128 patients who participated in the main study, 61 were in the navigation group, and 67 were in the usual care group. The response rate changed during the study period due to attrition. At the post-test timepoint, six patients in the navigation group and three in the usual care group were lost to follow-up. By the follow-up timepoint, an additional three patients in the navigation group and six in the usual care group were lost. The reasons for this loss to follow-up included 14 patient deaths, three withdrawals from the study, and one patient being physically unable to complete the questionnaires.

Homogeneity testing indicated no significant differences between the two groups regarding sociodemographic and disease-related variables (see Table 1 for the categorical variables and Table 2 for the continuous variables). The study population comprised patients with a variety of cancer types, namely breast, lung, colorectal, prostate, liver, oral, gynecological, and gastrointestinal cancers (including esophageal, stomach, and pancreatic cancers). Details on the distribution of and differences in all cancer types between the groups are provided in the Supplementary File 1. The inclusion of these specific types of cancer reflects their prevalence among the outpatient population involved in the study. Given the absence of significant differences in demographics or disease variables between the groups, no adjustments were made for potential confounding factors in the model. The baseline prevalence rates for anxiety, depression, emotional distress, and demoralization were 17.9%, 15.6%, 29.7%, and 29.7%, respectively.

### 3.2. Intervention Effects

**Figure 3:**
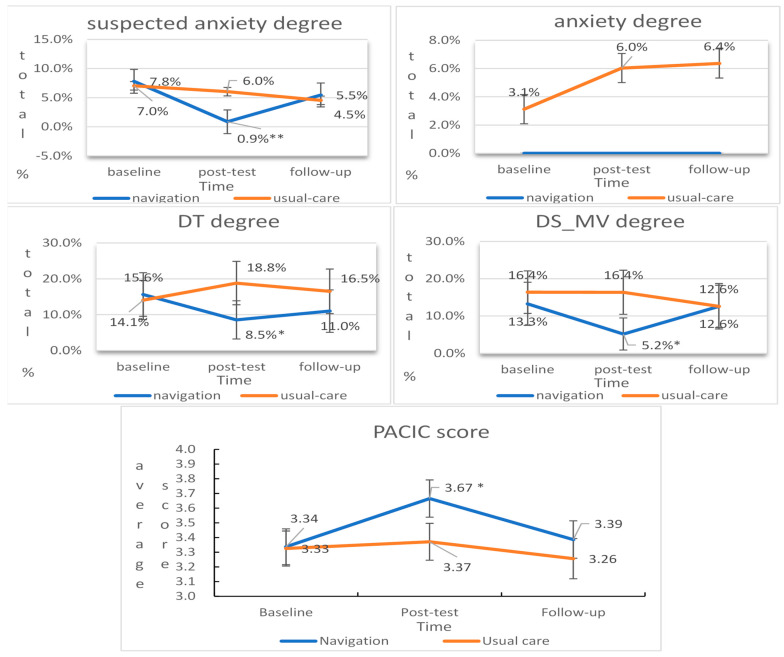
Changes in the prevalence rate of suspected anxiety, anxiety, DS_MV, DT, and PACIC between the two groups at each timepoint. * *p* < 0.05. ** *p* < 0.01.

Based on our GEE models, there was no significant difference in HADS-Depression results after three or six months of the intervention (Table 3). For the HADS-Anxiety results, although the navigation and usual care groups experienced increases in anxiety levels, these changes were not significantly different from the baseline at three (odds ratio (OR) = 1.26, 95% CI = 0.70–2.26, *p* = 0.441) or six months (OR = 1.32, 95% CI = 0.73–2.40, *p* = 0.357). However, there was a significant interaction effect between the navigation group and the post-test timepoint (*p* = 0.003), indicating that the navigation was more effective in reducing the likelihood of anxiety over this three-month period than usual care. Based on the estimated parameters, the OR between the two groups at three months was 0.08. This suggests that the odds of experiencing anxiety reduction were approximately 92% lower in the navigation group than in the usual care group at three months. The post-test prevalence rates for suspected anxiety were 0.9% and 6.0%, respectively. For the presence of anxiety, these rates were 0.0% and 6.0%, respectively (Figure 3).

For the binary variables from the DS_MV (cut-off ≥ 30 points) and the DT (cut-off ≥ 4 points), significant interaction effects were observed both between groups and with the post-test timepoint (*p* = 0.017 and *p* = 0.047), indicating that the likelihood of a reduction in demoralization and distress over three months was higher in the navigation group than in the usual care group, respectively. Based on the ORs, the odds of experiencing demoralization were approximately 75% lower in the navigation group (OR = 0.249), and those for experiencing distress were approximately 58% lower in the navigation group (OR = 0.415) than in the usual care group at three months. However, this difference was not observed at six months (Table 3). The post-test prevalence rates in the navigation and usual care groups were 5.2% and 16.4% for demoralization and 8.5% and 18.8% for distress, respectively (Figure 3).

For the PACIC score, there was also a significant interaction effect between the groups and the post-test timepoint (*p* = 0.020). The ORs indicated that the patients in the navigation group were approximately 1.40 times more likely to report a better patient experience with cancer care than those who received usual care (OR = 1.40) at three months (Table 3). The post-test mean PACIC scores were 3.37 and 3.67, respectively (Figure 3).

## 4. Discussion

### 4.1. Prevalence of Breast Cancer in Taiwan

Our study found that there were no significant differences in cancer diagnoses between the two groups at the baseline. However, the majority of the patients were affected by breast cancer (63.9% versus 65.7% in the navigation and usual care groups, respectively), reflecting the high incidence of this cancer type in our sample. Breast cancer significantly impacts public health in Taiwan, as evidenced by its high incidence and mortality rates. According to the Ministry of Health and Welfare, cancer has been the leading cause of death in Taiwan for 41 consecutive years, with breast cancer currently being the second leading cause of cancer deaths among women [42]. The incidence rate stands at 69.1 per 100,000 women, making breast cancer the most prevalent cancer among Taiwanese women, particularly those aged 45–69. Each year, over 10,000 new cases are diagnosed, and more than 2000 women die from breast cancer, which translates to approximately 31 diagnoses and 6 deaths per day [42,43]. This prevalence underlines the severity of breast cancer in Taiwan and sets the context for our study, which found a higher proportion of breast cancer participants.

Our study found that the participants in the navigation group exhibited significant improvements in anxiety, demoralization, emotional distress, and medical experience compared with those in the usual care group after three months. However, each group contained patients with both a high proportion of breast cancer and a small number of other cancer types. Therefore, a subgroup analysis for the effectiveness of navigation intervention between patients with breast cancer and other cancer types was conducted. Data analysis revealed no discernible differences in all study outcomes between the patients with breast cancer and those with other types of cancer (see Supplementary Table S1 and File S2 for details). This lack of variability suggests that the interventions, while standardized, may have masked potential benefits which more tailored approaches could reveal. Although the overall sample size was adequate, it may not have been large enough to detect small but clinically meaningful differences among various cancer types. Additionally, the measurement tools might not have been sensitive enough to distinguish the distinct impacts across different patient groups.

Yet, given the high prevalence and specific challenges of breast cancer in Taiwan, it is crucial to suggest the potential for more specialized, cancer type-specific interventions which might better address the particular biological and treatment-related complexities of breast cancer. Future research should consider the development of intervention strategies which are specifically designed for breast cancer care alongside those for other types of cancers and compare the effectiveness between the groups. This approach could help uncover whether more customized interventions could indeed yield different outcomes in breast cancer patients compared with those with other cancers. It may be beneficial to revisit the adequacy of the sample sizes for each cancer type subgroup and the sensitivity of the outcome measures used to ensure that they can capture subtle yet clinically important differences.

### 4.2. Mental Health Indicators (Primary Outcomes)

Consistent with previous studies [19,44], our investigation found that navigation can reduce anxiety in patients with cancer. Patients recently diagnosed with cancer and those planning to receive treatment have an urgent need for relevant and useful information, and this need often increases anxiety [45]. Anxiety is more common among patients with cancer than in relatively healthy people, especially since the cost of treatment is a major barrier [9]. The prevalence rate of anxiety of 17.9% in this study was similar to the 19.1% value reported by Naser et al. [46]. The proactive and supportive nature of navigation prevents feelings of isolation and helplessness at the end of life and provides channels for accessing resources, thus greatly reducing anxiety [47].

Regarding depression, there was no statistically significant difference between the navigation group and the usual care group. The results of our study differed from those of previous investigations [19,21], which recruited patients with breast cancer and an HADS depression score ≥ 8. A possible explanation for this may be that the proportion of patients with depression in this study was not high (15.7%), and it was only 5.7% (*N* = 7) in the navigation group. Nearly 80% of our participants in the present study had early-stage (0 or I) or intermediate-stage (II or III) cancer. Patients with advanced cancer are more likely to suffer from mental disorders and have poorer survival rates [48,49]. Thus, while the mental health status of our participants may have been suboptimal, it did not appear to include serious depression for the majority. Nonetheless, our findings were consistent with those of a recent randomized controlled trial [47].

Oncology nurse navigation in this study effectively provided immediate and individualized care. Prior to providing the intervention, they assessed the problems and needs of the patients using the DT scale. The prevalence rate of distress in this study was 29.7%, which was similar to that noted by Al-Shaaobi, Alahdal, Yu, and Pan [35] (28%) as well as Wang et al. [50] (33.2%) in local studies. According to the service process in this study, nurse navigators referred cancer patients with a DT score ≥ 4 to psychologists or psychiatrists. For patients who declined referrals, the navigation team provided additional assistance targeted toward overcoming their obstacles and enabling them to manage their distress and stress and adjust to their situations.

In our study, 29.7% of the patients had high levels of demoralization. This rate was consistent with that reported in a systematic review of patients with cancer (13.5–49.4%) [22]. Interventions targeted at improving symptoms of demoralization in patients with cancer can generally be divided into two categories: drug therapy and psychotherapy [51]. Nurse navigation includes drug education, communication, support, comprehensive integration of medical resources, and patient-centered care. Thus, it covers both of these categories [12,52]. It therefore makes sense that the navigation intervention had a significant effect on the demoralization of the patients in this study.

### 4.3. Patient Experience (Secondary Outcome)

As in previous research, this study showed that nurse navigation can improve patients’ experiences with medical services [18,19,53]. The terms “patient satisfaction” and “patient experience” are often used interchangeably in the literature. However, patient satisfaction reflects individual subjective expectations rather than the quality of the care itself and thus provides no insight into any root causes [54]. Patient experience can be defined as what happened during an episode of care and how it happened from the patient’s perspective. As such, research in recent years has focused mainly on this [55]. Patient experience is primarily the domain of interaction with healthcare, and it encompasses care structure, care process and outcomes, service utilization and access, and patient satisfaction [56]. Therefore, patient experience depends on effective communication, coordination, continuity, transition, and the appropriateness of personalized care provided by their medical service providers [52,56]. Previous randomized controlled trials and reviews suggest that improvements in patient experience as a result of navigation are related to the coordination and integration of medical resources, multidisciplinary care, assistance with overcoming barriers, and the provision of supportive care [9,16]. Furthermore, nurse navigation pre-appointment includes regular telephone follow-up services to relieve patients’ anxiety concerning their symptoms and medical treatment after their discharge from a hospital, as well as improving the patient experience [14,20]. These results suggest that an ONN program can potentially improve both the mental health and patient experience of cancer patients. Navigation is a form of patient-centered care. The navigator nurse helps patients with cancer overcome barriers and meet their goals through effective communication, comprehensive coordination, and the facilitation of interaction with other patients, families, and the healthcare system. Nurse navigator roles and programs should therefore be expanded across more healthcare systems. In addition, future research should investigate the qualitative aspects of the patient experience to enrich the quantitative findings of this study and further fine-tune the ONN intervention model.

### 4.4. Limitations

Firstly, conducting this study as a single-blinded control was challenging, because the study was conducted in an outpatient clinic area. There was no sure way of preventing patients in the usual care group from communicating with and consulting the navigator at any time, which may have interfered with the treatment’s effect. We therefore ensured that the navigators minimized their interactions with patients in the usual care group or delayed providing the service for six months.

Secondly, we excluded patients with preexisting psychiatric problems or receiving palliative or hospice care from the study to simplify and ensure the validity of our analyses. There-fore, the results were unable to be generalized across all patient categories. Future research should therefore investigate this effect in greater depth and use a wider spectrum of patients.

Third, there were zero cases of confirmed anxiety in the intervention group, which may have affected the fitting of and our ability to make inferences from the GEE model. Although some studies indicate that zero-case ordinal data have little impact on a GEE model [57,58], it is nevertheless important to interpret our results carefully. To address this issue, we conducted a sensitivity analysis to verify the stability of the findings by combining patients with suspected and confirmed anxiety (HADS-Anxiety score ≥ 8) into a binary outcome. This reanalysis also yielded a significant interaction effect between the groups and the post-test timepoint (*p* = 0.008, OR = 0.087, 95% CI = 0.014–0.534). The ordinal and binary analyses yielded consistent findings, indicating that the reduction in the likelihood of anxiety over a three-month period was greater in the navigation group than in the usual care group. This suggests that the model was fitted appropriately and capable of detecting significant differences despite the existence of zero confirmed anxiety cases in the intervention group.

Additionally, the intervals between the posttest and follow-up periods in this study (three and six months, respectively) might have been too short to observe significant within-group differences. Future studies should consider extending these periods to more than three and six months to potentially achieve significant outcomes within the groups. According to Llewellyn-Bennett et al. [59] and Fitzpatrick et al. [60], randomized clinical trials (RCTs) often have relatively short follow-up periods, which may underestimate the potential benefits of the treatment being studied and fail to detect risks which may take longer to emerge. Therefore, future research should explore longer follow-up durations to capture these potential effects more accurately. However, it is also important to note that extending the duration of RCTs could increase costs, and a balance must be struck between the benefits of longer follow-up periods and the associated costs.

### 4.5. Perspectives and Potential Applications

Our study highlights the importance of oncology nurse navigation in improving mental health outcomes, demonstrating its effectiveness in adult oncology care. However, the potential application of this intervention extends beyond adult patients. For instance, oncology nurse navigation could be applied to pediatric oncology patients and their families. Nurses play a crucial role in pediatric oncology, and their proactive engagement can significantly impact the mental health of both children and their families [61,62]. Similarly, the role of community health workers, as highlighted by Banayat et al. [63], is crucial in enhancing healthcare delivery by improving awareness and treatment adherence among underserved populations. Further supporting this approach, the Adolescent and Kids Advocacy Program (AKAP) pilot program, initiated in 2022, demonstrates the potential benefits of integrating strategic referral pathways to facilitate access to cancer clinical trials and comprehensive family support systems. In addition, education is crucial in training healthcare personnel. However, in low- and middle-income countries, its implementation is a luxury which hampers the improvement of medical standards. Interestingly, Banayat et al. [64] suggested using oncology website resources for learning, as while some sites are of low quality with limited knowledge, they overall represent a valuable channel for overcoming current barriers.

Despite the advancements, there are still relevant diagnostic barriers which may compromise survival in certain countries, particularly where access to modern tumor diagnostics and treatment is limited even in academic centers and not just in the low-income countries of Asia [65,66]. Future research should explore the adaptation of oncology nurse navigation programs to different patient demographics and healthcare environments, including low-resource settings, to maximize their impact. Other perspective points could include the integration of telehealth services to extend the reach of nurse navigation and the use of technology to enhance patient education and engagement [67]. Moreover, oncology nurse navigation can also be beneficial in addressing health disparities and improving outcomes for marginalized populations, including those in rural and resource-poor settings [68]. Effective navigation can ensure timely access to care, improve health equity, and support patients throughout the cancer continuum.

#### Novelty and Practical Impact

The novelty of this research lies in its comprehensive evaluation of the psychosocial impact of oncology nurse navigation across multiple dimensions of mental health. By incorporating a patient-centered approach, this study provides robust evidence supporting the integration of nurse navigators into standard oncology care. The practical impact of these findings is significant, suggesting that nurse navigators can play a crucial role in enhancing the quality of life for cancer patients and potentially leading to broader implementation of such programs in clinical settings globally.

## 5. Conclusions

This study demonstrates that oncology nurse navigation can significantly reduce anxiety, demoralization, and distress in cancer patients while improving their overall experience of care. The proactive coordination and communication facilitated by nurse navigators effectively bridge gaps between patients and healthcare providers, ensuring that patients receive timely referrals and appropriate services. These findings suggest that integrating nurse navigators into cancer care teams provides substantial psychosocial benefits, contributing to better mental health outcomes and patient experience.

## Figures and Tables

**Table 1:** Categorical variables of participant characteristics at baseline (*N* = 128).

Categorical Variable		Navigation Group (*n* = 61)	Usual Care Group (*n* = 67)	Fisher’s Exact Test *p*
		*n* (%)	*n* (%)	
Gender	Male	15 (24.6)	17 (25.4)	1.000
Marital status	Married or cohabiting	41 (67.2)	42 (62.7)	0.711
Education	College or higher	34 (55.7)	36 (53.7)	0.860
Household monthly income	USD ≤ 1540	22 (37.3)	26 (40.6)	0.957
	USD 1541–2156	14 (23.0)	13 (20.3)	
	USD ≥ 2157	23 (37.7)	25 (39.1)	
	Rejected	2 (3.3)	3 (4.5)	
Employment status	Yes	36 (59.0)	32 (47.8)	0.219
Religion	Yes	45 (73.8)	54 (80.6)	0.402
Exercise habits	Yes	42 (68.9)	48 (71.6)	0.847
Smoking status	Yes	15 (24.6)	13 (19.4)	0.525
Drinking habits	Yes	28 (45.9)	36 (53.7)	0.479
Areca (betel nut) use	Yes	4 (6.6)	5 (7.5)	1.000
Life dependence	Yes	4 (6.6)	6 (9.0)	0.747
Comorbidities	Yes	24 (39.3)	21 (31.3)	0.361
Family history of cancer	No	28 (45.9)	29 (43.3)	0.954
	Unknown	3 (4.9)	3 (4.5)	
	Yes	30 (49.2)	35 (52.2)	
Cancer diagnosis	Breast cancer	39 (63.9)	44 (65.7)	0.855
	Others ^a^	22 (36.1)	23 (34.3)	
Stage	Early (0, I)	23 (37.7)	27 (40.3)	0.973
	Intermediate (II, III)	25 (41.0)	26 (38.8)	
	Severe (IV)	13 (21.3)	14 (20.9)	
Treatment	Chemotherapy	12 (19.7)	13 (19.4)	0.913
	Radiotherapy	15 (24.6)	14 (20.9)	
	Other	34 (55.7)	40 (59.7)	
DT	<4	41 (67.2)	49 (73.1)	0.562
	≥4	20 (32.8)	18 (26.9)	
HADS_D	Non-depression (≤7)	54 (88.5)	54 (80.6)	0.335
	Suspected (8–10)	5 (8.2)	11 (16.4)	
	Depression (≥11)	2 (3.3)	2 (3.0)	
HADS_A	Non-anxiety (≤7)	51 (83.6)	54 (80.6)	0.177
	Suspected (8–10)	10 (16.4)	9 (13.4)	
	Anxiety (≥11)	0 (0)	4 (6.0)	
DS_MV	<30	44 (72.1)	46 (68.7)	0.702
	≥30	17 (27.9)	21 (31.3)	

Note on abbreviations: DT = Distress Thermometer; HADS_A = Hospital Anxiety and Depression Scale—Anxiety; HADS_D = Hospital Anxiety and Depression Scale—Depression; DS_MV = Demoralization Scale (Mandarin Version); USD = United States dollar. ^a^ Others = colorectal cancer, lung cancer, liver cancer, oral cancer (including nasopharyngeal cancer), prostate cancer, urological cancers (including kidney cancer), stomach cancer, pancreatic cancer, gynecological cancers (including uterine and ovarian cancer), esophageal cancer, lymphoma, and osteosarcoma.

**Table 2:** Continuous variables of participant characteristics at baseline (*N* = 128).

Continuous Variable	Navigation Group (*n* = 61)	Usual Care Group (*n* = 67)	*p*
	Mean/Median (*SD*/*IQR*)	Mean/Median (*SD*/*IQR*)	
Age	54.2 (11.3)	56.7 (12.8)	0.258
PACIC	3.3 (1.0)	3.3 (1.0)	0.995
DS_MV	22.4 (11.6)	24.1 (10.9)	0.388
Time since diagnosis (yrs) ^†^	0.6 (2.5)	0.4 (1.2)	0.190
DT (baseline) ^†^	2.0 (3.5)	2.0 (3.0)	0.766
HADS_A (baseline) ^†^	5.0 (2.5)	6.0 (4.0)	0.488
HADS_D (baseline) ^†^	3.0 (4.0)	4.0 (3.0)	0.081

Note on abbreviations: DS_MV = Demoralization Scale (Mandarin Version); DT = Distress Thermometer; HADS_A = Hospital Anxiety and Depression Scale—Anxiety; HADS_D = Hospital Anxiety and Depression Scale—Depression; NTD = New Taiwan Dollar; PACIC = Patient Assessment of Chronic Illness Care; yrs = years; SD = standard deviation; IQR = interquartile ranges. ^†^ Non-normal distribution (Kolmogorov–Smirnov test) using the nonparametric Mann–Whitney U test.

**Table 3:** Analysis of intervention effects with generalized estimating equations.

Variable	HADS-Anxiety (Category)					PACIC (Continuous)				
	*B*	*SE*	*p*	*OR*	95% CI for *OR*	*B*	*SE*	*p*	*OR*	95% CI for *OR*
Group										
Navigation	−0.24	0.45	0.598	0.79	0.32–1.92	<−0.01	0.17	0.995	1.00	0.72–1.39
Usual care	Reference									
Time										
Follow-up	0.28	0.30	0.357	1.32	0.73–1.40	−0.09	0.12	0.450	0.91	0.72–1.16
Posttest	0.23	0.30	0.441	1.26	0.70–2.26	0.01	0.12	0.940	1.01	0.80–1.27
Baseline	Reference									
Interaction ((Group) × (Time))										
(Navigation) × (Follow-up)	−0.68	0.57	0.230	0.51	0.23–0.51	0.18	0.17	0.290	1.20	0.86–1.68
(Navigation) × (Posttest)	−2.25	0.76	0.003	0.11	0.02–0.47	0.35	0.15	0.020	1.42	1.06–1.91
Reference: (Navigation) × (Baseline), (Usual care) × (Follow-up), (Usual care) × (Posttest), (Usual care) × (Baseline)										
**Variable**	**DT (Binary)**					**DS_MV (Binary)**				
	***B***	***SE***	***p***	***OR***	**95% CI** **for** ***OR***	***B***	***SE***	***p***	***OR***	**95% CI** **for** ***OR***
Group										
Navigation	0.28	0.39	0.465	1.33	0.62–2.84	−0.17	0.39	0.668	0.85	0.40–1.81
Usual care	Reference									
Time										
Follow	0.23	0.33	0.474	1.26	0.67–2.40	−0.31	0.32	0.336	0.74	0.39–1.38
Posttest	0.39	0.37	0.295	1.47	0.72–3.02	0.01	0.34	0.966	1.01	0.52–1.97
Baseline	Reference									
Interaction ((Group) × (Time))										
(Navigation) × (Follow)	−0.72	0.52	0.168	0.49	0.18–1.35	0.27	0.45	0.549	1.31	0.54–3.20
(Navigation) × (Posttest)	−1.16	0.58	0.047	0.31	0.10–.98	−1.22	0.52	0.017	0.29	0.11–0.81
Reference: (Navigation) × (Baseline), (Usual care) × (Follow), (Usual care) × (Posttest), (Usual care) × (Baseline)										

Note on abbreviations: *B* = beta coefficient; *SE* = standard error; *p* = *p* value; CI = confidence intervals; *OR* = odds ratio; HADS_A = Hospital Anxiety and Depression Scale—Anxiety; DT = Distress Thermometer; DS_MV = Demoralization Scale (Mandarin Version); PACIC = Patient Assessment of Chronic Illness Care.

## Data Availability

Data of our study are unavailable due to privacy and ethical restrictions.

## Supplementary Materials

The following supporting information can be downloaded at: https://www.mdpi.com/article/10.3390/curroncol31070306/s1, Table S1: Outcome Comparison: Breast Cancer vs. Others. File S1: All cancer types. File S2: Statistics.

## References

[1] Sung H., Ferlay J., Siegel R.L., Laversanne M., Soerjomataram I., Jemal A., Bray F. (2021). Global cancer statistics 2020: Globocan estimates of incidence and mortality worldwide for 36 cancers in 185 countries. CA A Cancer J. Clin..

[2] World Health Organization [WHO] Partnering to Improve the Quality of Cancer Care: Who Teams Up with the World’s Leading Organization for Physicians and Oncology Professionals. https://www.who.int/news/item/04-06-2022-partnering-to-improve-the-quality-of-cancer-care-who-teams-up-with-the-worlds-leading-organization-for-physicians-and-oncology-professionals.

[3] Chiang C.-J., Lo W.-C., Yang Y.-W., You S.-L., Chen C.-J., Lai M.-S. (2016). Incidence and survival of adult cancer patients in taiwan, 2002–2012. J. Formos. Med. Assoc..

[4] Mehta R.D., Roth A.J. (2015). Psychiatric considerations in the oncology setting. CA A Cancer J. Clin..

[5] Linden W., Vodermaier A., Mackenzie R., Greig D. (2012). Anxiety and depression after cancer diagnosis: Prevalence rates by cancer type, gender, and age. J. Affect. Disord..

[6] Zahid J.A., Grummedal O., Madsen M.T., Gogenur I. (2020). Prevention of depression in patients with cancer: A systematic review and meta-analysis of randomized controlled trials. J. Psychiatr. Res..

[7] Lang-Rollin I., Berberich G. (2018). Psycho-oncology. Dialogues Clin. Neurosci..

[8] Freeman H.P., Rodriguez R.L. (2011). History and principles of patient navigation. Cancer.

[9] Chan R.J., Milch V.E., Crawford-Williams F., Agbejule O.A., Joseph R., Johal J., Dick N., Wallen M.P., Ratcliffe J., Agarwal A. (2023). Patient navigation across the cancer care continuum: An overview of systematic reviews and emerging literature. CA A Cancer J. Clin..

[10] Leahy D., Donnelly A., Irwin K., D’Alton P. (2021). Barriers and facilitators to accessing cancer care for people with significant mental health difficulties: A qualitative review and narrative synthesis. Psycho-Oncology.

[11] Anderson J.E., Larke S.C. (2009). Navigating the mental health and addictions maze: A community-based pilot project of a new role in primary mental health care. Ment. Health Fam. Med..

[12] Oncology Nursing Society [ONS] 2017 Oncology Nurse Navigator Core Competencies. https://www.ons.org/sites/default/files/2017ONNcompetencies.pdf.

[13] Oncology Nursing Society [ONS] Role of the Oncology Nurse Navigator throughout the Cancer Trajectory. https://www.ons.org/make-difference/advocacy-and-policy/position-statements/ONN.

[14] Newton J.C., O’Connor M., Saunders C., Ali S., Nowak A.K., Halkett G.K. (2022). “Who can I ring? Where can I go?” living with advanced cancer whilst navigating the health system: A qualitative study. Support. Care Cancer.

[15] Oh J., Ahn S. (2021). Effects of nurse navigators during the transition from cancer screening to the first treatment phase: A systematic review and meta-analysis. Asian Nurs. Res..

[16] Kokorelias K.M., Shiers-Hanley J.E., Rios J., Knoepfli A., Hitzig S.L. (2021). Factors influencing the implementation of patient navigation programs for adults with complex needs: A scoping review of the literature. Health Serv. Insights.

[17] Winterhalter M.P., Cobb K., Heron K., Metzgar M., McCullough C., Rux S. (2023). Nurse navigators as the drivers to an enhanced patient referral process. J. Oncol. Navig. Surviv..

[18] Ludman E.J., McCorkle R., Bowles E.A., Rutter C.M., Chubak J., Tuzzio L., Jones S., Reid R.J., Penfold R., Wagner E.H. (2015). Do depressed newly diagnosed cancer patients differentially benefit from nurse navigation?. Gen. General. Hosp. Psychiatry.

[19] Mertz B.G., Dunn-Henriksen A.K., Kroman N., Johansen C., Andersen K.G., Andersson M., Mathiesen U.B., Vibe-Petersen J., Dalton S.O., Envold Bidstrup P. (2017). The effects of individually tailored nurse navigation for patients with newly diagnosed breast cancer: A randomized pilot study. Acta Oncol..

[20] Wang T., Huilgol Y.S., Black J., D’Andrea C., James J., Northrop A., Belkora J.K., Esserman L.J. (2021). Pre-appointment nurse navigation: Patient-centered findings from a survey of patients with breast cancer. CJON.

[21] Bidstrup P.E., Johansen C., Kroman N., Belmonte F., Duriaud H., Dalton S.O., Andersen K.G., Mertz B. (2023). Effect of a nurse navigation intervention on mental symptoms in patients with psychological vulnerability and breast cancer: The rebecca randomized clinical trial. JAMA Netw. Open.

[22] Wang Y., Sun H., Ji Q., Wu Q., Wei J., Zhu P. (2023). Prevalence, associated factors and adverse outcomes of demoralization in cancer patients: A decade of systematic review. Am. J. Hosp. Palliat. Med..

[23] Lee S.C.K., Wang C.H., Hsieh C.I., Su L.Y., Chi C.L., Lu C.L. (2016). Patient navigation for cancer support: The cancer resource center in taiwan. J. Oncol. Nurs..

[24] Kelly K.J., Doucet S., Luke A. (2019). Exploring the roles, functions, and background of patient navigators and case managers: A scoping review. Int. J. Nurs. Stud..

[25] Schutt R.K., Siegfriedt J., Fawcett J. (2017). Who cares? Case management and patient navigation in a public health programme. Int. J. Care Caring.

[26] Cancer Research UK What Do Cancer Stages and Grades Mean?. https://www.nhs.uk/common-health-questions/operations-tests-and-procedures/what-do-cancer-stages-and-grades-mean/.

[27] Compton C.C., Byrd D.R., Garcia-Aguilar J., Kurtzman S.H., Olawaiye A., Washington M.K. (2012). AJCC Cancer Staging Atlas: A Companion to the Seventh Editions of the AJCC Cancer Staging Manual and Handbook.

[28] Zigmond A.S., Snaith R.P. (1983). The hospital anxiety and depression scale. Acta Psychiatr. Scand..

[29] Lee Y., Wu Y.S., Chien C.Y., Fang F.M., Hung C.F. (2016). Use of the hospital anxiety and depression scale and the taiwanese depression questionnaire for screening depression in head and neck cancer patients in taiwan. Neuropsychiatr. Dis. Treat..

[30] Roth A.J., Kornblith A.B., Batel-Copel L., Peabody E., Scher H.I., Holland J.C. (1998). Rapid screening for psychologic distress in men with prostate carcinoma: A pilot study. Cancer.

[31] Donovan K.A., Handzo G., Corbett C., Vanderlan J., Brewer B.W., Ahmed K. (2022). Nccn distress thermometer problem list update. J. Natl. Compr. Cancer Netw..

[32] Sun H., Thapa S., Wang B., Fu X., Yu S. (2021). A systematic review and meta-analysis of the distress thermometer for screening distress in asian patients with cancer. J. Clin. Psychol. Med. Settings.

[33] Smith R., Mannle S.E., Livsey K.R., Tait E., Rossitch J.C. (2017). Where fear begins: The effect of a nurse navigator home visit to decrease distress in newly diagnosed breast cancer patients. J. Oncol. Navig. Surviv..

[34] Tang L.-l., Zhang Y.-n., Pang Y., Zhang H.-w., Song L.-l. (2011). Validation and reliability of distress thermometer in chinese cancer patients. Chin. J. Cancer Res..

[35] Al-Shaaobi A., Alahdal M., Yu S., Pan H. (2021). The efficiency of distress thermometer in the determination of supporting needs for cancer inpatients. Libyan J. Med..

[36] Kissane D.W., Wein S., Love A., Lee X.Q., Kee P.L., Clarke D.M. (2004). The demoralization scale: A report of its development and preliminary validation. J. Palliat. Care.

[37] Cheng J., Chen J., Zhang Y., Kissane D., Yan J. (2019). Translation and psychometric properties for the demoralization scale in chinese breast cancer patients. Eur. J. Oncol. Nurs..

[38] Center for Accelerating Care Transformation [ACT Center] Translations and Adaptations. https://www.act-center.org/our-work/primary-care-transformation/assessments/translations-and-adaptations.

[39] Glasgow R.E., Wagner E.H., Schaefer J., Mahoney L.D., Reid R.J., Greene S.M. (2005). Development and validation of the patient assessment of chronic illness care (PACIC). Med. Care.

[40] Li C.S. (2020). The Effectiveness of Nursing Navigation on Psychological Distress, Anxiety and Depression and Demoralization among Patients with Cancer: A Pilot Study.

[41] Feise R.J. (2002). Do multiple outcome measures require *p*-value adjustment?. BMC Med. Res. Methodol..

[42] Ministry of Health and Welfare 2023 Cause of Death Statistics. https://dep.mohw.gov.tw/DOS/lp-5069-113-xCat-y112.html.

[43] Ministry of Health and Welfare Breast Cancer Prevention and Treatment. https://www.hpa.gov.tw/Pages/Detail.aspx?nodeid=614&pid=1124.

[44] Harding M.M. (2015). Effect of nurse navigation on patient care satisfaction and distress associated with breast biopsy. Clin. J. Oncol. Nurs..

[45] Nikbakhsh N., Moudi S., Abbasian S., Khafri S. (2014). Prevalence of depression and anxiety among cancer patients. Casp. J. Intern. Med..

[46] Naser A.Y., Hameed A.N., Mustafa N., Alwafi H., Dahmash E.Z., Alyami H.S., Khalil H. (2021). Depression and anxiety in patients with cancer: A cross-sectional study. Front. Psychol..

[47] Soto-Perez-de-Celis E., Chavarri-Guerra Y., Ramos-Lopez W.A., Alcalde-Castro J., Covarrubias-Gomez A., Navarro-Lara Á., Quiroz-Friedman P., Sánchez-Román S., Alcocer-Castillejos N., Aguilar-Velazco J.C. (2021). Patient navigation to improve early access to supportive care for patients with advanced cancer in resource-limited settings: A randomized controlled trial. Oncology.

[48] Koo M.M., Swann R., McPhail S., Abel G.A., Elliss-Brookes L., Rubin G.P., Lyratzopoulos G. (2020). Presenting symptoms of cancer and stage at diagnosis: Evidence from a cross-sectional, population-based study. Lancet Oncol..

[49] Davis L.E., Bogner E., Coburn N.G., Hanna T.P., Kurdyak P., Groome P.A., Mahar A.L. (2020). Stage at diagnosis and survival in patients with cancer and a pre-existing mental illness: A meta-analysis. J. Epidemiol. Community Health.

[50] Wang G.L., Cheng C.T., Feng A.C., Hsu S.H., Hou Y.C., Chiu C.Y. (2016). Prevalence, risk factors, and the desire for help of distressed newly diagnosed cancer patients: A large-sample study. Palliat. Support. Care.

[51] Wang Y., Sun H., Ji Q., Wei J., Zhu P. (2023). Systematic review of interventions for demoralization in patients with cancer. J. Nerv. Ment. Dis..

[52] Berkowitz B. (2016). The patient experience and patient satisfaction: Measurement of a complex dynamic. Online J. Issues Nurs..

[53] Berezowska A., Passchier E., Bleiker E. (2021). Professional patient navigation in a hospital setting: A randomized controlled trial. Support. Care Cancer.

[54] Anufriyeva V., Pavlova M., Stepurko T., Groot W. (2021). The validity and reliability of self-reported satisfaction with healthcare as a measure of quality: A systematic literature review. Int. J. Qual. Health Care.

[55] Bull C., Byrnes J., Hettiarachchi R., Downes M. (2019). A systematic review of the validity and reliability of patient-reported experience measures. Health Serv. Res..

[56] Bourque M.A., Loiselle C.G. (2022). Patients’ cancer care perceptions conceptualized through the cancer experience measurement framework. BMC Health Serv. Res..

[57] Chen Y., Cao Z., Li J., Chen J., Zhu Q., Liang S., Lan G., Xing H., Liao L., Feng Y. (2023). Hiv transmission and associated factors under the scale-up of hiv antiretroviral therapy: A population-based longitudinal molecular network study. Virol. J..

[58] Fitzmaurice G., Davidian M., Verbeke G., Molenberghs G. (2008). Generalized estimating equations for longitudinal data analysis. Longitudinal Data Analysis.

[59] Llewellyn-Bennett R., Edwards D., Roberts N., Hainsworth A.H., Bulbulia R., Bowman L. (2018). Post-trial follow-up methodology in large randomised controlled trials: A systematic review. Trials.

[60] Fitzpatrick T., Perrier L., Shakik S., Cairncross Z., Tricco A.C., Lix L., Zwarenstein M., Rosella L., Henry D. (2018). Assessment of long-term follow-up of randomized trial participants by linkage to routinely collected data: A scoping review and analysis. JAMA Netw. Open.

[61] Jong M., Lown E.A., Schats W., Mills M.L., Otto H.R., Gabrielsen L.E., Jong M.C. (2021). A scoping review to map the concept, content, and outcome of wilderness programs for childhood cancer survivors. PLoS ONE.

[62] Schuster M.A. (2021). Creating the hematology/oncology/stem cell transplant advancing resiliency team: A nurse-led support program for hematology/oncology/stem cell transplant staff. J. Pediatr. Oncol. Nurs..

[63] Banayat A.C., Abad P.J.B., Bonito S.R., Manahan L.T., Peralta A.B. (2023). Care needs of parents of children with cancer in a low-middle-income country. J. Pediatr. Hematol./Oncol. Nurs..

[64] Banayat A.C., Challinor J., Sniderman E. (2023). An expert evaluation of oncology website resources for use in pediatric oncology clinical nursing education in low-resource settings. J. Pediatr. Hematol./Oncol. Nurs..

[65] World Health Organization [WHO] Global Burden of Mental Disorders and the Need for a Comprehensive, Coordinated Response from Health and Social Sectors at the Country Level. http://apps.who.int/gb/ebwha/pdf_files/EB130/B130_9-en.pdf.

[66] National Cancer Institute Cancer Disparities. https://www.cancer.gov/about-cancer/understanding/disparities.

[67] Budde H., Williams G.A., Scarpetti G., Kroezen M., Maier C.B. What Are Patient Navigators and How Can They Improve Integration of Care?. https://apps.who.int/iris/rest/bitstreams/1404871/retrieve.

[68] Flucke N. (2021). Patient assessment: Using the oncology nurse navigator patient assessment for rural and other resource-poor settings. Clin. J. Oncol. Nurs..

