# Supplementary material for: The Effect of Oncology Nurse Navigation on Mental Health in Patients with Cancer in Taiwan: A Randomized Controlled Clinical Trial
Source: Curr Oncol. 2024 Jul 20;31(7):4105–22. doi: 10.3390/curroncol31070306 (PMC11276177; doi:10.3390/curroncol31070306)
Supplement: Supplementary file 1 [file curroncol-31-00306-s001.zip › File S1. all cancer types.pdf]

Category Variable		Navigation group (N = 61) n (%)	Usual-care group (N = 67) n (%)	χ^2	p
Cancer diagnosis	Breast cancer	39 (63.9)	44 (65.7)	5.09	.964[a]
	Colorectal cancer	6 (9.8)	3 (4.5)		
	Lung cancer	3 (4.9)	3 (4.5)		
	Liver cancer	2 (3.3)	2 (3.0)		
	Oral cancer (including nasopharyngeal cancer)	3 (4.9)	3 (4.5)		
	Prostate cancer and urological cancers (including kidney cancer)	2 (3.3)	4 (6.0)		
	Stomach cancer and pancreatic cancer	3 (4.9)	3 (4.5)		
	Gynecological cancers (including uterine and ovarian cancer)	1 (1.6)	1 (1.5)		
	Esophageal cancer	0 (0.0)	1 (1.5)		
	Lymphoma	1 (1.6)	3 (4.5)		
	Osteosarcoma	1 (1.6)	0 (0.0)		

[a] Fisher's exact test: 20 cells (90.9%) had an expected count less than 5.
