# Supplementary material for: The Effect of Oncology Nurse Navigation on Mental Health in Patients with Cancer in Taiwan: A Randomized Controlled Clinical Trial
Source: Curr Oncol. 2024 Jul 20;31(7):4105–22. doi: 10.3390/curroncol31070306 (PMC11276177; doi:10.3390/curroncol31070306)
Supplement: Supplementary file 1 [file curroncol-31-00306-s001.zip › File S2. statistics.pdf]

$$Y = a_0 + a_1X + a_2T_1 + a_3T_2 + b_1XT_1 + b_2XT_2$$

		T0	T1	T2
X=gp	1	$a_0 + a_1$	~~a_0~~ +-a_1 + ~~a_2~~-+ b_1	~~a_0~~ + a_1 + ~~a_3~~ + b_2
	0	a_1	~~a_0~~ + ~~a_2~~	~~a_0~~ + ~~a_3~~
			1 vs. 0	1 vs. 0
			$e^{a1 + b1}$	$e^{a1 + b2}$
			$e^{(-0.24-2.25)}$	$e-^{(0.24-0.68)}$

a_1=-0.24, a_2=0.23, a_3=0.28, b1=-2.25, b_2=-0.68
