# Supplementary material for: The Effect of Oncology Nurse Navigation on Mental Health in Patients with Cancer in Taiwan: A Randomized Controlled Clinical Trial
Source: Curr Oncol. 2024 Jul 20;31(7):4105–22. doi: 10.3390/curroncol31070306 (PMC11276177; doi:10.3390/curroncol31070306)
Supplement: Supplementary file 1 [file curroncol-31-00306-s001.zip › Table S1.pdf]

Table S1. Outcome Comparison: Breast Cancer vs. Others

**Variable**	**HADS-anxiety (Category)**					**HADS-depression (Category)**				
	B	***SE***	***p***	***OR***	***95% CI for OR***	***B***	***SE***	***p***	***OR***	***95% CI for OR***
Group										
Breast	-1.14	0.62	.066	0.32	0.09-1.08	0.07	0.51	.898	1.07	0.39-2.89
Others	Reference									
Time										
Follow	-0.42	0.59	.480	0.66	0.21-2.10	-0.37	0.48	.447	0.69	0.27-1.79
Post-test	-0.38	0.60	.525	0.68	0.21-2.22	0.41	0.57	.477	1.50	0.49-4.58
Baseline	Reference									
Interaction										
(Group) × (Time)										
(Breast) × (Follow)	0.41	0.67	.542	1.51	0.40-5.61	0.39	0.60	.513	1.48	0.46-4.75
(Breast) × (Post-test)	0.69	0.68	.310	1.99	0.53-7.49	-0.48	0.64	.450	0.62	0.18-2.16
Reference: (Breast) × (Baseline), (Others) × (Follow), (Usual-care) × (Post-test), (Others) × (Baseline)										

**Variable**	**DT (Binary)**					**DS_MV (Binary)**				
	B	*SE*	*p*	*OR*	95% CI for *OR*	*B*	*SE*	*p*	*OR*	95% CI for *OR*
Group										
Breast	0.59	0.43	0.171	1.799	0.78-4.18	-0.57	0.42	0.180	0.57	0.25-1.30
Usual-care	Reference									
Time										
Follow	0.10	0.45	0.824	1.106	0.46-2.69	-0.20	0.42	0.643	0.82	0.36-1.88
Post-test	0.03	0.52	0.954	1.031	0.37-2.88	0.64	0.53	0.225	1.90	0.68-5.34
Baseline	Reference									
Interaction										
[Group) × (Time)										
(Breast) × (Follow)	-0.31	0.55	0.573	0.733	0.25-2.16	0.57	0.50	0.256	1.76	0.66-4.69
(Breast) × (Post-test)	-0.21	0.62	0.732	0.808	0.24-2.74	-0.26	0.60	0.662	0.77	0.24-2.50
Reference: (Breast) × (Baseline), (Others) × (Follow), (Others) × (Post-test), (Others) × (Baseline)										

**Variable**	**PACIC (Continuous)**				
	B	*SE*	*p*	*OR*	95% CI for *OR*
Group					
Breast	0.05	0.18	0.793	1.00	0.74-1.49
Others	Reference				

Time					
Follow	0.16	0.15	0.269	0.91	0.88-1.56
Post-test	0.25	0.14	0.073	1.01	0.98-1.70
Baseline			Reference		
Interaction					
(Group) × (Time)					
(Breast) × (Follow)	0.27	0.45	0.549	1.31	0.54-3.20
(Breast) × (Post-test)	-1.22	0.52	0.017	0.29	0.11-0.81

Reference: (Breast) × (Baseline), (Others) × (Follow), (Others) × (Post-test), (Others) × (Baseline)
